# Genome-wide association study on Fourier transform infrared milk spectra for two Danish dairy cattle breeds

**DOI:** 10.1186/s12863-020-0810-4

**Published:** 2020-01-31

**Authors:** R. M. Zaalberg, L. Janss, A. J. Buitenhuis

**Affiliations:** Department of Molecular Biology and Genetics, Center for Quantitative Genetics and Genomics, Aarhus University, DK-8830 Tjele, Denmark

**Keywords:** Spectroscopy, Genetic architecture, Breed difference, Infra-red

## Abstract

**Background:**

Infrared spectral analysis of milk is cheap, fast, and accurate. Infrared light interacts with chemical bonds present inside the milk, which means that Fourier transform infrared milk spectra are a reflection of the chemical composition of milk. Heritability of Fourier transform infrared milk spectra has been analysed previously. Further genetic analysis of Fourier transform infrared milk spectra could give us a better insight in the genes underlying milk composition. Breed influences milk composition, yet not much is known about the effect of breed on Fourier transform infrared milk spectra. Improved understanding of the effect of breed on Fourier transform infrared milk spectra could enhance efficient application of Fourier transform infrared milk spectra. The aim of this study is to perform a genome wide association study on a selection of wavenumbers for Danish Holstein and Danish Jersey. This will improve our understanding of the genetics underlying milk composition in these two dairy cattle breeds.

**Results:**

For each breed separately, fifteen wavenumbers were analysed. Overall, more quantitative trait loci were observed for Danish Jersey compared to Danish Holstein. For both breeds, the majority of the wavenumbers was most strongly associated to a genomic region on BTA 14 harbouring *DGAT1.* Furthermore, for both breeds most quantitative trait loci were observed for wavenumbers that interact with the chemical bond C-O. For Danish Jersey, wavenumbers that interact with C-H were associated to genes that are involved in fatty acid synthesis, such as *AGPAT3*, *AGPAT6*, *PPARGC1A*, *SREBF1*, and *FADS1*. For wavenumbers which interact with –OH, associations were observed to genomic regions that have been linked to alpha-lactalbumin.

**Conclusions:**

The current study identified many quantitative trait loci that underlie Fourier transform infrared milk spectra, and thus milk composition. Differences were observed between groups of wavenumbers that interact with different chemical bonds. Both overlapping and different QTL were observed for Danish Holstein and Danish Jersey.

## Background

There is a large number of applications for trait predictions utilizing Fourier transform infrared (FT-IR) milk spectra from the mid-infrared range. Fourier-transform infrared spectroscopy determines light absorbance across the infrared spectrum. Light is absorbed when it interacts with chemical bonds. Wavenumber 1690 cm^− 1^, for example, interacts with C=O of amide I, and 1600 cm^− 1^ is involved in N-H bending of amide II [1, 2]. These chemical bonds are typical for protein molecules. Wavenumbers from the lower energy region that ranges from 1150 to 1040 cm^− 1^ interact with C-OH, which is abundantly present in sugar molecules [1, 2]. This chemical bond, however, is also present in fat and protein molecules, but more scarcely. Wavelengths from the infrared region that ranges from 2950 to 2850 cm^− 1^ induce C-H stretching [1, 2]. Triglyceride molecules are rich in C-H bonds, but C-H bonds are also present in many other molecules.

Mid-infrared light is commonly used in combination with the principal least square regression method to analyse chemical composition of milk [3]. The major milk components fat, protein, and lactose have been successfully predicted with FT-IR milk spectra [3]. In addition, minor milk components have been predicted with FT-IR milk spectra, such as fatty acids [4–6], protein fractions [7, 8], and ketone bodies [9–11]. Concentration of ketone bodies in milk can be used as an indicator for subclinical ketosis [9–11], or energy balance [12, 13].

Associations to genomic regions have been observed for both milk composition, and infrared milk spectra. Fatty acid composition, for example, has been associated to many different genomic regions [14, 15]. FT-IR milk spectra have been linked to genes that have been associated to milk composition previously, such as *Diacylglycerol O-acyltransferase 1* (***DGAT1***) or *beta-lactoglobulin* (***PAEP***) [16–18]. A genome wide association study (**GWAS**) on a subset of wavenumbers revealed associations for individual wavenumbers to a variety of genes [18]. Examples are the gene for the growth hormone receptor, or the gene *UMPS* [18]. FT-IR milk spectra are also moderately to strongly heritable [17, 19–21]. To get a better understanding of the genetic background of FT-IR milk spectra, it is necessary to further study the association between milk spectra and the genome.

Cattle breed influences milk composition [19, 22–24], and the genetic architecture of milk composition [25–27]. These breed differences in milk composition are reflected in the FT-IR milk spectra. Heritability of FT-IR milk spectra varied across breeds [17, 19–21]. Not much is known about the breed differences in the genes that indirectly underlie FT-IR milk spectra. Enhanced knowledge on breed differences in the genetic architecture of FT-IR milk spectra could provide us with a better understanding of differences in milk composition across breeds. Finally, it could facilitate future application of FT-IR milk spectra in across breed prediction of novel phenotypes.

The aim of this study is to perform a GWAS on a selection of wavenumbers in two dairy cattle breeds, Danish Holstein and Danish Jersey.

## Results

### Selection of wavenumbers

**Fig. 1:**
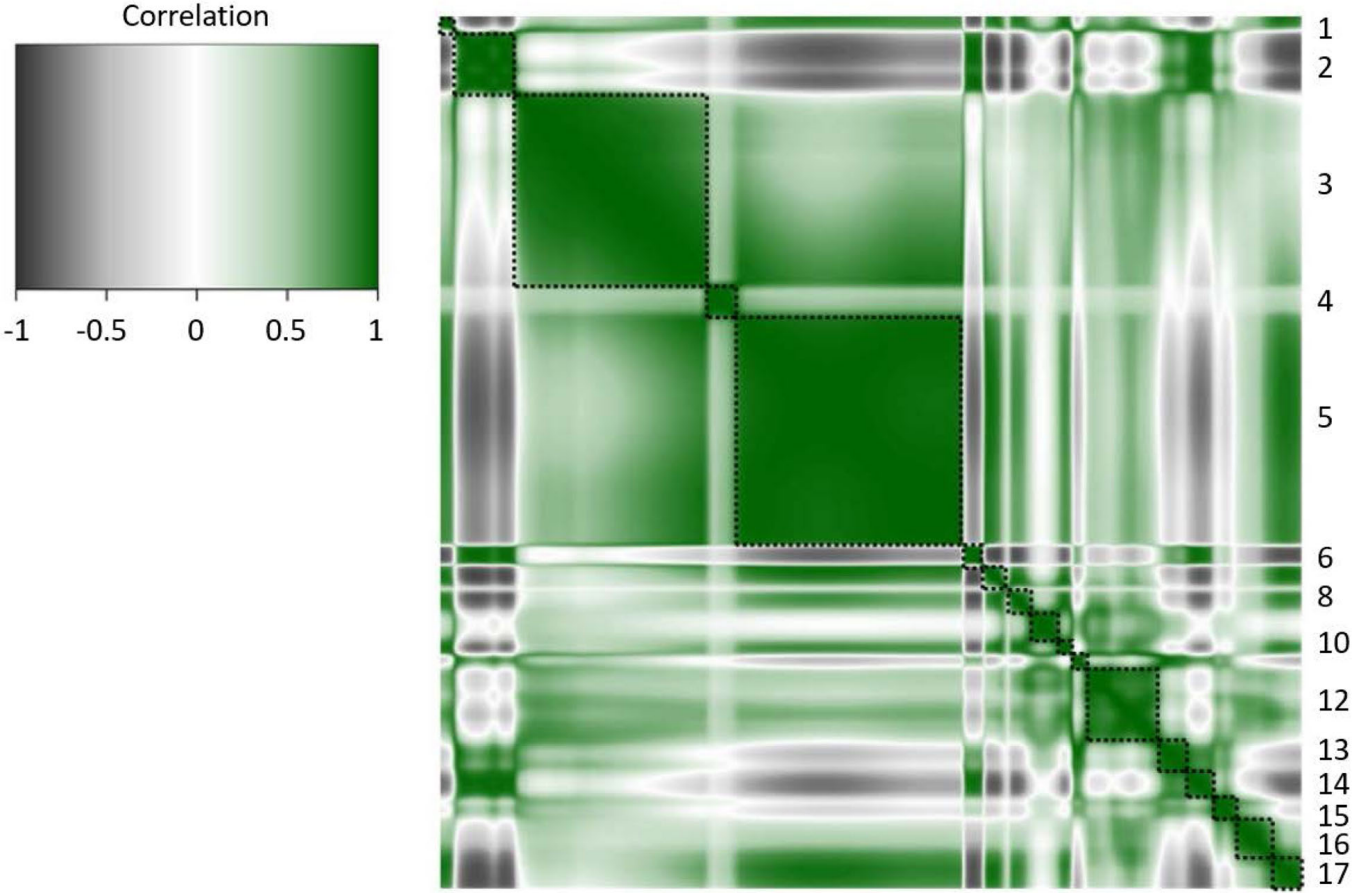
Heatmap of the phenotypic correlation matrix for wavenumbers in Danish Holstein. The upper and lower triangle are identical. Seventeen blocks of strongly positively correlated neighbouring wavenumbers are indicated with black dashed square boxes. The upper left corner represents wavenumber 3008 cm^− 1^ and wavenumber group 1, and the lower right represents wavenumber 925 cm^− 1^ and wavenumber group 17

After removal of wavenumbers which interact with water molecules, 530 waveunumbers were left. For these 530 wavenumbers, correlations were calculated. The correlation matrices were nearly identical for Danish Holstein and Danish Jersey. The heatmap of the correlation matrix for Danish Holstein is presented in Fig. 1. For both breeds 17 blocks of highly correlated neighbouring wavenumbers were observed, where the correlation between wavenumbers was > 0.95. From each block, the wavenumber with the highest correlation sum was selected. For four blocks, the selected wavenumber was different for Danish Holstein and Danish Jersey (Table 1). For both Danish Holstein and Danish Jersey, 15 out of the 17 selected wavenumbers had a heritability > 0.05. For Danish Holstein and Danish Jersey separately, Table 1 presents an overview of the selected wavenumber per block, the chemical bond with which the wavenumber interacts, heritability of the selected wavenumber, number of quantitative trait loci (**QTL**) identified for the selected wavenumber, number of QTL unique for the selected wavenumber, and chromosomes on which QTL were located. A QTL was defined as one, or several overlapping groups of 100 neighbouring SNPs (**SNP group**), for which each individual SNP group explained > 0.35% of the total additive genetic variation. A peak was defined as the SNP group within a QTL, which explained most of the total additive genetic variation.

**Table 1:** Fifteen selected wavenumbers (wvn) from fifteen positively correlated wavenumber blocks (see Fig. 1), the chemical bond (CB) with which the selected wavenumber interacts, heritability of the selected wavenumber, total number of QTL for the selected wavenumber, number of QTL unique for the selected wavenumber, and chromosomes on which QTL were located

**Danish Holstein**						
**Block**	**Wvn (cm**^**− 1**^**)**	**CB**^**a**^	***h***^***2***^	**# QTL**	**# Unique QTL**	**BTA**
1	2988	C-H	0.14	5	–	5, 14, 17, 20, 21
2	2872	C-H	0.25	2	–	5, 14
5	1966	Unknown	0.16	3	–	5, 14, 21
6	1735	C=O	0.21	3	–	5, 14, 17
7	1696	C=O	0.18	2	–	5, 14
8	1604	N-H	0.20	4	–	5, 14, 17, 20
9	1557	N-H	0.18	3	–	6, 14, 20
10	1500	CO-N	0.12	3	–	5, 6, 14
11	1449	Alkanes	0.26	6	1	6, 11, 13, 14, **19**, 29
12	1295	-OH	0.11	5	–	14, 19, 20, 21, 29
13	1226	C-O	0.26	5	–	3, 5, 11, 14, 29
14	1180	C-O	0.24	3	–	5, 14, 17
15	1114	C-O	0.28	7	–	3, 11, 14, 19, 25, 28, 29
16	1060	C-O	0.20	5	–	3, 14, 19, 21, 29
17	975	C-O	0.17	3	–	5, 14, 21
**Danish Jersey**						
**Block**	**Wvn (cm**^**−1**^**)**	**CB**	***h***^***2***^	**# QTL**	**# Unique QTL**	**BTA**
1	2988	C-H	0.12	10	1	1, 5, 6, 11, 14, 19, 20, 23, **27**, 29
2	2872	C-H	0.22	16	–	1, 5, 6, 8, 9, 11, 12, 14, 16, 17, 18, 19, 20, 21, 24, 25
5	1966	Unknown	0.15	12	1	3, 5, 6, 9, **11**, 12, 14, 16, 19, 20, 21, 22
6	1735	C=O	0.18	6	–	5, 11, 12, 14, 19, 20
7	1696	C=O	0.15	12	–	3, 5, 6, 9, 11, 12, 14, 16, 19, 20, 21, 22
8	1604	N-H	0.12	19	4	1, **2**, 3, **5**, 6, **7**, 8, 9, 10, 11, 12, 14, 16, 19, **20**, 22, 23, 24
9	1557	N-H	0.15	12	4	**1**, **2**, 3, 5, 6, **7**, 11, 13, 16, **18**, 20, 29
10	1500	CO-N	0.06	12	2	6, 10, 11, 12, 14, 16, **17**, 20, **21**, 23, 24, 29
11	1449	Alkanes	0.25	12	–	3, 5, 6, 11, 12, 14, 16, 18, 19, 20, 21, 25
12	1299	-OH	0.07	10	1	1, 5, 6, 14, 16, 18, 19, 23, **27**, 28
13	1226	C-O	0.20	17	1	1, 5, 6, **8**, 9, 11, 12, 14, 16, 17, 18, 19, 20, 21, 22, 25, 28
14	1183	C-O	0.20	17	1	1, 5, **6**, 8, 9, 11, 12, 14, 16, 17, 18, 19, 20, 21, 22, 25
15	1114	C-O	0.20	14	4	1, **2**, 4, 5, 6, **7**, **10**, 14, 16, **17**, 18, 19, 25, 28
16	1056	C-O	0.19	4	–	1, 14, 16, 19
17	979	C-O	0.19	9	–	3, 5, 9, 11, 12, 14, 16, 19, 20

Boldface chromosomes are those where unique QTL were observed

^a^Williams and Fleming, 1980 [2]

### Peak regions

Table 2 shows an overview of genomic regions, which were associated to groups of wavenumbers, which interact with different chemical bonds. For each group of wavenumbers, genomic regions of 100 consecutive SNPs which explained > 0.35% of the total additive genetic variation are listed. This genomic region is referred to as the “peak region”. There can be more peak regions on one chromosome. Table 2 gives an overview of the highest peak region for each chromosome, meaning that only one peak region per chromosome is described. A peak region is not necessarily associated to all wavenumbers of a group. For each peak region, those wavenumbers are presented for which the proportion of explained additive genetic variation by the peak region > 0,35%. Candidate genes located within the peak region are named in the final column.

**Table 2:** Top SNP groups explaining most total additive genetic variation for wavenumbers, which interact with different chemical bonds

**Danish Holstein**							
**Chemical bond**	**Peak region**					**All associated wvn**	**pVarA**
	**Peak**	**CHR**	**LL**	**UL**	**Candidate genes**		
Alkanes	6_b	6	80394422	88006286	*CSN*-cluster	**1449**	0.54
	11_a	11	69240871	75495975		**1449**	0.4
	13_a	13	53205470	58871642		**1449**	0.37
	14	14	1463676	5428037	*DGAT1*	**1449**	2.04
	19_a	19	32148966	37552530	*SREBF1*	**1449**	0.39
	29_a	29	6893054	12147907		**1449**	0.48
C=O	5_b	5	91118692	98515360	*MGST1*	**1735**, **1696**	0.43
	14	14	1463676	5428037	*DGAT1*	**1735**, 1696	9.76
	17_a	17	12545331	18398611		**1735**	0.39
C-H	5_b	5	91118692	98515360	*MGST1*	2988, **2872**	0.39
	14	14	1463676	5428037	*DGAT1*	2988, **2872**	7.88
	17_a	17	12545331	18398611		**2988**	0.36
	20_b	20	56721394	61566803	*ANKH*	**2988**	0.36
	21_a	21	6241052	11219294	*IGF1R*	**2988**	0.36
C-O	3_c	3	49704834	58434544		1226, **1114**, 1060	0.42
	5_b	5	91118692	98515360	*MGST1*	1226, **1180**, 975	0.47
	11_a	11	69240871	75495975		1226, **1114**	0.43
	14	14	1463676	5428037	*DGAT1*	1226, **1180**, 1114, 1060, 975	8.55
	17_a	17	12545331	18398611		**1180**	0.37
	19_c	19	57592897	62235799		1226, 1114, **1060**	0.52
	21_a	21	6241052	11219294	*IGF1R*	1226, 1114, **1060**	0.35
	21_c	21	62530384	67845758		**975**	0.35
	25	25	47181	4689960		1226, 1180, **1114**, 1060, 975	0.37
	28_a	28	2313753	7557315		1226, 1180, **1114**, 1060, 975	0.39
	29_a	29	6893054	12147907		1226, **1114**, 1060	0.54
CO-N	5_b	5	94381154	101721892	*MGST1*	**1500**	0.36
	6_b	6	86819633	92465869	*CSN*-cluster	**1500**	0.35
	14	14	1463676	5428037	*DGAT1*	**1500**	5.19
N-H	5_b	5	91118692	98515360	*MGST1*	**1604**	0.5
	6_b	6	80394422	88006286	*CSN*-cluster	**1557**	0.55
	14	14	1463676	5428037	*DGAT1*	**1604**, 1557	9.73
	17_a	17	12545331	18398611		**1604**	0.37
	20_b	20	54443531	59156949	*ANKH*	**1604**, 1557	0.53
-OH	14	14	1463676	5428037	*DGAT1*	**1295**	1.12
	19_c	19	58318121	63354178		**1295**	0.38
	20_b	20	56721394	61566803	*ANKH*	**1295**	0.4
	21_a	21	6241052	11219294	*IGF1R*	**1295**	0.36
	29_a	29	6893054	12147907		**1295**	0.45
**Danish Jersey**							
**Chemical bond**	**Peak**					**All associated wvn**	**pVarA**
	**Peak**	**CHR**	**LL**	**UL**	**Candidate gene(s)**		
Alkanes	3_a	3	10619258	19193451	*GBA*	**1449**	0.57
	5_a	5	69483211	76659850		**1449**	0.6
	6_b	6	81119938	88592295	*CSN*-cluster	**1449**	2.25
	11_b	11	101802657	106804258	*PAEP*	**1449**	0.48
	12_b	12	67073994	78212571		**1449**	0.47
	14	14	1463676	5601692	*DGAT1*	**1449**	2.1
	16_a	16	37904090	46625869		**1449**	0.54
	18_b	18	29414941	37188964		**1449**	0.41
	19_a	19	31087581	36437188	*SREBF1*	**1449**	0.55
	20_a	20	28803514	35940949	*GHR*, *MRPS30*	**1449**	0.67
	21_b	21	53369113	61180711	*PI*	**1449**	0.4
	25	25	1127441	5948405		**1449**	0.35
C=O	3_b	3	31035370	37101347		1735, **1696**	0.35
	5_a	5	72082602	79640097		**1735**, **1696**	0.42
	6_a	6	41496235	46788536	*PPARGC1A*	**1696**	0.4
	9_a	9	123525	5981648		1735, **1696**	0.39
	11_b	11	100858404	105845271	*PAEP*	**1735**, 1696	0.44
	12_b	12	67073994	78212571		1735, **1696**	0.54
	14	14	1463676	5601692	*DGAT1*	**1735**, 1696	5.11
	16_a	16	40846801	48806575		**1696**	0.36
	19_a	19	32148966	37582865	*SREBF1*	1735, **1696**	0.54
	20_a	20	28803514	35940949	*GHR*, *MRPS30*	1735, **1696**	0.6
	21_b	21	52091583	59665710	*PI*	**1696**	0.39
	22_a	22	162018	5884558		1735, **1696**	0.37
C-H	1_b	1	143831554	148893434	*SLC37A1, AGPAT3*	**2988**, 2872	0.62
	5_a	5	70897603	77988054		2988, **2872**	0.46
	6_a	6	41496235	46788536	*PPARGC1A*	**2988**, 2872	0.47
	8_a	8	50575791	55477297		2988, **2872**	0.35
	9_a	9	123525	5981648		2988, **2872**	0.39
	11_b	11	100858404	105845271	*PAEP*	2988, **2872**	0.44
	12_b	12	67073994	78212571		2988, **2872**	0.63
	14	14	1463676	5601692	*DGAT1*	2988, **2872**	5.38
	16_a	16	44176019	51011081		2988, **2872**	0.37
	17_c	17	66304866	71192864		**2872**	0.36
	18_a	18	162653	6934625		2988, **2872**	0.4
	19_a	19	32148966	37582865	*SREBF1*	2988, **2872**	0.62
	20_a	20	28803514	35940949	*GHR*, *MRPS30*	2988, **2872**	0.64
	21_b	21	53369113	61180711	*PI*	2988, **2872**	0.46
	23	23	33645739	40993370	*PRL*	**2988**, 2872	0.37
	24	24	31982958	37002274		2988, **2872**	0.35
	25	25	1127441	5948405		2988, **2872**	0.38
	27_b	27	32800374	38436906	*AGPAT6*	**2988**, 2872	0.37
	29_b	29	40344120	45817015	*FADS1*	**2988**, 2872	0.35
C-O	1_b	1	141807977	146940031	*SLC37A1, AGPAT3*	1226, **1183**	0.71
	2_a	2	1826336	8188132		1226, 1183, **1114**, 1056, 979	0.41
	3_a	3	15658202	24149985	*GBA*	1226, 1114, **979**	0.38
	4	4	116209319	120641946		**1114**	0.43
	5_a	5	70897603	77988054		1226, 1183, 1114, 1056, **979**	0.51
	6_b	6	81119938	88592295	*CSN*-cluster	**1226**, 1183, 1114	0.88
	7_a	7	244816	5027447		1226, 1183, **1114**, 1056, 979	0.41
	8_b	8	102776706	108556349		**1226**, 1183, 979	0.38
	9_a	9	123525	5981648		**1226**, 1183, 1114, 1056, 979	0.37
	9_b	9	23322608	29910981		1226, 1183, 1114, **979**	0.37
	10_a	10	26527257	32295516		1226, 1183, **1114**, 979	0.37
	11_b	11	100858404	105845271	*PAEP*	1226, **1183**, 979	0.51
	12_b	12	67073994	78212571		1226, **1183**, 1114, 1056, 979	0.67
	14	14	1463676	5601692	*DGAT1*	1226, **1183**, 1114, 1056, 979	5.27
	16_b	16	66365432	71735866		1226, 1183, 1114, **1056**, 979	0.43
	17_c	17	67193210	72005076		1226, **1183**, 1114, 1056, 979	0.38
	17_c	17	66304866	71192864		**1226**, 1183, 1114, 1056, 979	0.38
	18_a	18	162653	6934625		1226, **1183**, 1114, 1056, 979	0.41
	18_b	18	32335701	38197798		**1226**, 1183, 1114, 979	0.41
	19_a	19	31087581	36437188	*SREBF1*	1226, 1183, **1114**, 979	0.68
	19_b	19	48007453	53568928	*GH*, *FASN*, *CCDC57*	**1056**, 979	0.66
	20_a	20	28803514	35940949	*GHR*, *MRPS30*	1226, **1183**, 1114, 979	0.63
	21_b	21	53369113	61180711	*PI*	**1226**, 1183, 1114, 1056, 979	0.46
	22_b	22	47619247	54132903	*LTF*	**1226**, 1183, 1114, 1056, 979	0.43
	25	25	47181	4952802		1226, 1183, **1114**, 1056, 979	0.39
	25	25	1127441	5948405		**1226**, 1183, 1114, 1056, 979	0.39
	28_b	28	17171751	23666465		1226, **1114**, 979	0.47
CO-N	6_b	6	81119938	88592295	*CSN*-cluster	**1500**	1.04
	10_b	10	49435552	54012929		**1500**	0.37
	11_b	11	101802657	106804258	*PAEP*	**1500**	0.41
	12_a	12	15817583	23340411		**1500**	0.39
	14	14	1463676	5601692	*DGAT1*	**1500**	1.9
	16_a	16	40846801	48806575		**1500**	0.44
	17_a	17	42892776	47808938		**1500**	0.42
	20_a	20	27422842	34474873	*GHR*, *MRPS30*	**1500**	0.36
	21_a	21	38947	8955497	*IGF1R*	**1500**	0.35
	23	23	28021047	35406968	*PRL*	**1500**	0.43
	24	24	33000605	37958693		**1500**	0.35
	29_b	29	40344120	45817015	*FADS1*	**1500**	0.49
N-H	1_a	1	68997018	76331663		**1604**, 1557	0.4
	2	2	108823285	114517270		**1557**	0.36
	2	2	27374270	31731639		**1604**, 1557	0.36
	3	3	10619258	19193451	*GBA*	1604, **1557**	0.73
	5_a	5	69483211	76659850		1604, **1557**	0.65
	6_b	6	81119938	88592295	*CSN*-cluster	1604, **1557**	2.32
	7_a	7	5955666	16564424		**1604**, 1557	0.36
	7_b	7	73511189	78936990		1604, **1557**	0.36
	8_a	8	50575791	55477297		**1604**	0.35
	9_a	9	123525	5981648		**1604**	0.38
	10_b	10	48475000	52884624		**1604**	0.36
	11_b	11	101802657	106804258	*PAEP*	**1604**, **1557**	0.45
	12_a	12	15817583	23340411		**1604**, 1557	0.44
	13_b	13	74795835	79730805		1604, **1557**	0.35
	14	14	1463676	5601692	*DGAT1*	**1604**	4.89
	16_a	16	37003329	44151335		1604, **1557**	0.49
	18_a	18	8061697	14038121		1604, **1557**	0.49
	19_a	19	31087581	36437188	*SREBF1*	**1604**, 1557	0.38
	20_a	20	27422842	34474873	*GHR*, *MRPS30*	1604, **1557**	0.52
	20_b	20	55992767	60817895	*ANKH*	**1604**	0.49
	22_a	22	162018	5884558		**1604**	0.36
	23	23	28021047	35406968	*PRL*	**1604**, 1557	0.4
	24	24	29942533	34992601		**1604**, 1557	0.36
	29_b	29	40344120	45817015	*FADS1*	1604, **1557**	0.41
-OH	1_b	1	142832701	147841620	*SLC37A1, AGPAT3*	**1299**	0.39
	5_b	5	87904220	94858411	*MGST1*	**1299**	0.38
	6_b	6	81119938	88592295	*CSN*-cluster	**1299**	1.09
	14	14	1463676	5601692	*DGAT1*	**1299**	0.52
	16_a	16	40846801	48806575		**1299**	0.46
	18_b	18	27578257	35583594		**1299**	0.36
	19_b	19	49037959	54488129	*FASN*, *CCDC57*	**1299**	0.42
	23	23	38521106	44428685		**1299**	0.36
	27_a	27	20369666	25633356		**1299**	0.35
	28_b	28	17171751	23666465		**1299**	0.39

The table shows wavenumbers for which the peak SNP-group explained > 0.35% of total additive genetic variation, the upper-, and lower limit of the peak SNP-group, and candidate genes which have been previously associated to milk composition traits. Wavenumbers in boldface are those with the highest percentage total additive genetic variation explained by the peak SNP-group

An overview of the number of QTL per chromosome, for Danish Holstein and Danish Jersey separately, and the number of overlapping QTL between the two breeds are shown in Table 3. Results are presented for all wavenumbers combined, and for groups of wavenumbers based on the chemical bond with which they interact (Table 1).

**Table 3:** Number of QTL per chromosome observed for Danish Holstein (DH) and Danish Jersey (DJ), for wavenumbers which interact with different chemical bonds. Overlap (ol) indicates number of overlapping QTL between the two breeds

	Total			Wavenumbers interacting with^a^																				
				*Alkanes*			*C-O*			*CO-N*			*N-H*			*C=O*			*C-H*			*-OH*		
CHR	*DH*	*DJ*	*ol*^a^	*DH*	*DJ*	*ol*	*DH*	*DJ*	*ol*	*DH*	*DJ*	*ol*	*DH*	*DJ*	*ol*	*DH*	*DJ*	*ol*	*DH*	*DJ*	*ol*	*DH*	*DJ*	*ol*
1	–	5	–	–	–	–	–	2	–	–	–	–	–	2	–	–	–	–	–	2	–	–	1	–
2	–	3	–	–	–	–	–	1	–	–	–	–	–	2	–	–	–	–	–	–	–	–	–	–
3	1	4	–	–	1	–	1	1	–	–	–	–	–	2	–	–	1	–	–	–	–	–	–	–
4	–	1	–	–	–	–	–	1	–	–	–	–	–	–	–	–	–	–	–	–	–	–	–	–
5	2	8	2	–	1	–	1	3	1	1	–	–	1	2	–	1	2	–	1	2	–	–	1	–
6	2	3	2	1	1	1	–	3	–	1	1	1	1	1	1	–	1	–	–	2	–	–	1	–
7	–	3	–	–	–	–	–	1	–	–	–	–	–	2	–	–	–	–	–	–	–	–	–	–
8	–	2	–	–	–	–	–	2	–	–	–	–	–	1	–	–	–	–	–	1	–	–	–	–
9	–	2	–	–	–	–	–	2	–	–	–	–	–	1	–	–	1	–	–	1	–	–	–	–
10	–	3	–	–	–	–	–	1	–	–	1	–	–	1	–	–	–	–	–	–	–	–	–	–
11	1	4	–	1	1	–	1	2	–	–	1	–	–	1	–	–	2	–	–	2	–	–	–	–
12	–	4	–	–	1	–	–	2	–	–	1	–	–	1	–	–	2	–	–	1	–	–	–	–
13	1	1	–	1	–	–	–	–	–	–	–	–	–	1	–	–	–	–	–	–	–	–	–	–
14	1	1	1	1	1	1	1	1	1	1	1	1	1	1	1	1	1	1	1	1	1	1	1	1
15	–	–	–	–	–	–	–	–	–	–	–	–	–	–	–	–	–	–	–	–	–	–	–	–
16	–	8	–	–	1	–	–	5	–	–	1	–	–	2	–	–	1	–	–	1	–	–	1	–
17	1	4	–	–	–	–	1	3	–	–	1	–	1	–	–	1	–	–	1	1	–	–	–	–
18	–	5	–	–	1	–	–	3	–	–	–	–	–	1	–	–	–	–	–	1	–	–	1	–
19	3	6	1	1	1	1	1	3	–	–	–	–	–	1	–	–	1	–	–	2	–	1	1	–
20	3	4	3	–	1	–	–	1	–	–	1	–	2	3	2	–	1	–	1	1	–	1	–	–
21	2	3	1	–	1	–	2	1	–	–	1	–	–	–	–	–	1	–	1	1	–	1	–	–
22	–	3	–	–	–	–	–	1	–	–	–	–	–	1	–	–	1	–	–	–	–	–	–	–
23	–	3	–	–	–	–	–	–	–	–	1	–	–	1	–	–	–	–	–	1	–	–	1	–
24	–	3	–	–	–	–	–	–	–	–	1	–	–	1	–	–	–	–	–	1	–	–	–	–
25	1	3	2	–	1	–	1	2	2	–	–	–	–	–	–	–	–	–	–	1	–	–	–	–
26	–	–	–	–	–	–	–	–	–	–	–	–	–	–	–	–	–	–	–	–	–	–	–	–
27	–	2	–	–	–	–	–	–	–	–	–	–	–	–	–	–	–	–	–	1	–	–	1	–
28	1	2	–	–	–	–	1	2	–	–	–	–	–	–	–	–	–	–	–	–	–	–	1	–
29	1	1	–	1	–	–	1	–	–	–	1	–	–	1	–	–	–	–	–	1	–	1	–	–
Total	20	91	11	6	12	3	11	43	4	3	12	2	6	29	4	3	15	1	5	24	1	5	10	1

^a^*Alkanes* 1449 cm^−1^; *C-O* 1226–975 cm^−1^; *CO-N*: 1500 cm^−1^; *N-H*: 1604–1557 cm^−1^; *C=O*: 1735–1696 cm^−1^; *C-H*: 2988–2872 cm^−1^; *−OH*: 1299–1295 cm^−1^

### QTL and wavenumbers interacting with different chemical bonds

#### Wavenumbers interacting with alkanes

For Danish Holstein, the three peak regions explaining most additive genetic variation for wavenumbers interacting with alkanes were positioned on BTA 6 (0.54%) harbouring the *casein* (***CSN***) cluster, on BTA 14 (2.04%), and on BTA 29 (0.48%). For Danish Jersey, the three peak regions were positioned on BTA 6 (2.25%) harbouring the *CSN* cluster, on BTA 14 (2.10%), and on BTA 20 (0.67%) harbouring *GHR*, and *MRPS30*. The *CSN* cluster is a genomic region on BTA 6 containing genes, which code for the milk protein casein.

#### Wavenumbers interacting with C=O

For Danish Holstein, the three peak regions explaining most additive genetic variation for wavenumbers interacting with C=O were positioned on BTA 5 (0.43%) harbouring *MGST1*, on BTA 14 (9.76%), and on BTA 17 (0.39%). For Danish Jersey, the four peak regions were positioned on BTA 12 (0.54%), on BTA 14 (5.11%), on BTA 19 (0.54%) harbouring *SREBF1*, and on BTA 20 (0.60%) harbouring *GHR*, and *MRPS30*.

#### Wavenumbers interacting with C-H

For Danish Holstein, the two peak regions explaining most additive genetic variation for wavenumbers interacting with C-H were positioned on BTA 5 (0.39%) harbouring *MGST1*, and on BTA 14 (7.88%). For Danish Jersey, the three peak regions were positioned on BTA 12 (0.63%), on BTA 14 (5.38%), and on BTA 20 (0.64%) harbouring *GHR*, and *MRPS30*.

#### Wavenumbers interacting with C-O

For Danish Holstein, the three peak regions explaining most additive genetic variation for wavenumbers interacting with C-O were positioned on BTA 14 (8.55%), on BTA 19 (0.52%), and on BTA 29 (0.54%). For Danish Jersey, the four peak regions were positioned on BTA 1 (0.71%), and *AGPAT3*, on BTA 6 (0.88) harbouring the *CSN* cluster, on BTA 14 (5.27%), and on BTA 19 (0.68%) harbouring *SREBF1*.

#### Wavenumbers interacting with CO-N

For Danish Holstein, the three peak regions explaining most additive genetic variation for wavenumbers interacting with C-ON were positioned on BTA 5 (0.36%) harbouring *MGST1*, on BTA 6 (0.35%) harbouring the *CSN* cluster, and on BTA 14 (5.19%). For Danish Jersey, the three peak regions were positioned on BTA 6 (1.04%) harbouring the *CSN* cluster, on BTA 14 (1.90%), and on BTA 29 (0.49%) harbouring *FADS1*.

#### Wavenumbers interacting with N-H

For Danish Holstein, the three peak regions explaining most additive genetic variation for wavenumbers interacting with N-H were positioned on BTA 6 (0.55%) harbouring the *CSN* cluster, on BTA 14 (9.73%), and on BTA 20 (0.53%) harbouring *ANKH*. For Danish Jersey, the three peak regions were positioned on BTA 3 (0.73%) harbouring *GBA*, on BTA 6 (2.32%) harbouring the *CSN* cluster, and on BTA 14 (4.89%).

#### Wavenumbers interacting with –OH

For Danish Holstein, the three peak regions explaining most additive genetic variation for wavenumbers interacting with -OH were positioned on BTA 14 (1.12%), on BTA 20 (0.40%) harbouring *ANKH*, and on BTA 29 (0.45%). For Danish Jersey, the three peak regions were positioned on BTA 6 (1.09%) harbouring the *CSN* cluster, on BTA 14 (0.52%), and BTA 16 (0.46%).

### Breed differences

Breed differences are clearly visible in Tables 1, 2 and 3, and the Manhattan plots in Additional files 1 and 2. Overall, more QTL were observed for Danish Jersey compared to Danish Holstein (Tables 2 and 3). For Danish Holstein, most QTL were located on BTA 19 and BTA 20, and for Danish Jersey on BTA 5 and BTA 16. For both breeds, most QTL were observed for wavenumbers interacting with C-O. Heritability of wavenumbers was slightly lower for Danish Jersey than for Danish Holstein (Table 1). The proportion of explained variation by the peak region of *DGAT1* was higher in Danish Holstein compared to Danish Jersey.

#### Overlapping QTL

Overlapping peak regions were observed on BTA 5 (91.1–94.9 Mbp) harbouring *MGST1*, on BTA 6 (81.1–84.6 Mbp) harbouring the *CSN* cluster, on BTA 19 (32.1–37.6), on BTA 20 (56.0–60.8 Mbp) harbouring *ANKH*, on BTA 21 (6.2–11.0 Mbp) harbouring *IGFIR*, and on BTA 25 (0.1–4.7 Mbp). Most overlapping QTL were observed for wavenumbers interacting with C-O and N-H. No overlapping QTL between the two breeds were observed for wavenumbers interacting with C=O, C-H, or –OH.

## Discussion

To get a better understanding of the genetics of milk composition, this study aimed at performing a GWAS on a selection of wavenumbers interacting with different chemical bonds in two dairy cattle breeds, Danish Holstein and Danish Jersey.

For each breed separately, fifteen wavenumbers were selected from blocks of strongly positively correlated neighbouring wavenumbers based on the maximum correlation sum within block, and a minimum heritability of 0.05. The correlation between wavenumbers within one block were close to one, and analysis of all wavenumbers within one block would most probably result in similar findings. For four blocks, different wavenumbers were selected for Danish Holstein and Danish Jersey (Table 1). The selected wavenumbers were within the same infrared region. Therefore, we assumed that results of e.g. 1295 cm^− 1^ for Danish Holstein are comparable to results of 1299 cm^− 1^ for Danish Jersey.

### BTA 14

A QTL on *Bos Taurus* autosome (**BTA**) 14 in the genomic region of *DGAT1* was associated to all wavenumbers, with the exception of 1557 cm^− 1^ in Danish Jersey. The QTL in *DGAT1* explained most additive genetic variation for 14 out of 15 wavenumbers in Danish Holstein, and for 9 out of 15 wavenumbers in Danish Jersey (Table 2)*.* Because *DGAT1* is a well-known major milk gene, the genomic region of BTA 14 will not be thoroughly discussed.

### Wavenumbers interacting with alkanes

The wavenumber 1449 cm^− 1^ is known to interact with alkanes [1, 2]. The chemical bonds present in alkanes resemble those of saturated fatty acids. For both breeds, a QTL on BTA 19 (19_a) was identified. This genomic region harbours the gene *SREBF1*. The gene *SREBF1* is known as a key player in fatty acid synthesis [14]. In line with this, Bouwman et al. [28] observed a QTL for saturated fatty acids in milk in the same genomic region. For both Danish Holstein and Danish Jersey, a QTL on BTA 6 (6_b) harbouring the *CSN* cluster was observed. This QTL has previously been associated to protein percentage [27, 29–31], caseins, whey proteins [30], and cheese yield [32]. In a GWAS on wavenumbers in Dutch Friesian Holstein, Wang and Bovenhuis [18] also observed an association between the QTL 6_b and wavenumber 1469 cm^− 1^.

### Wavenumbers interacting with C=O

The chemical bond C=O typically appears in fat molecules and protein molecules, and interacts with 1735 and 1696 cm^− 1^ [1, 2]. The QTL associated to wavenumbers interacting with C=O have been associated to a variety of milk production traits. In Danish Holstein, a QTL on BTA 5 (5_a) harbouring *MGST1* has previously been associated to milk composition [29, 30, 33], and fatty acid composition [28, 34]. In Danish Jersey, the QTL on BTA 6 (6_a) harbouring *PPARGC1A*, and BTA 19 (19_a) harbouring *SREBF1* have both been associated to fat percentage, and fatty acid composition in milk [14, 28, 34, 35]. Furthermore, for Danish Jersey, several QTL were identified that were linked to protein in milk previously. A QTL on BTA 11 (11_b) harbouring *PAEP* has been strongly associated to beta-lactoglobulin in milk, and protein composition [27, 30].

### Wavenumbers interacting with C-H

The chemical bond C-H is present in many molecules, such as fat, protein, and lactose. The C-H bond strongly interacts with 2988 and 2872 cm^− 1^ [1, 2]. The C-H bond is most abundantly present in the fatty acid tails of fat molecules. This is why wavenumbers in the region of 2988 and 2872 cm^− 1^ are used for prediction of fat percentage in milk [1, 36]. In Danish Holstein, the QTL on BTA 5 (5_b) harbouring *MGST1*, and the QTL on BTA 17 (17_a) have been associated to fatty acid composition in milk previously [28, 34]. For Danish Jersey, many QTL were located in genomic regions of genes, which have been associated to milk fatty acid synthesis [14, 37]. Examples of these genes are *AGPAT3* on BTA 1 (1_b), *PPARGC1A* on BTA 6 (6_a), *SREBF1* on BTA 19 (19_a), *AGPAT6* on BTA 27 (27_b), and *FADS1* on BTA 29 (29_b) [14, 37]. The gene *AGPAT6* on BTA 27 is described as one of the key links in milk fatty acid synthesis [37]. Interestingly, the genomic region of *AGPAT6* was only associated to wavenumbers that interact with C-H (Table 2). An additional QTL on BTA 20 (20_b) harbouring *ANKH* was observed for Danish Holstein. This QTL has been strongly associated to alpha-lactalbumin [27], and lactose percentage in milk [18]. For Danish Jersey, two QTL (11_b and 21_b) were found. Within this genomic region, two genes were located that have been linked to proteins in milk [27, 30, 38].

### Wavenumbers interacting with C-O

The chemical bond C-O is abundantly present in sugar molecules, and it interacts with wavenumbers in the infrared region from 1250 to 950 cm^− 1^ [1, 2]. This infrared region and the infrared region that ranges from 1400 to 1250 cm^− 1^ (see next section) are used for prediction of lactose in milk [1, 36]. For Danish Holstein, the observed QTL did not reveal a strong link between this infrared region and lactose in milk. The QTL on BTA 5 (5_b) harbouring *MGST1*, however, has been associated to milk composition, including lactose percentage [29]. Most of the QTL observed for Danish Holstein, however, have been associated to fatty acids or groups of fatty acids, such as the QTL on BTA 17 (17_a), BTA 19 (19_c), BTA 21 (21_a and 21_c), and BTA 28 (28_a) [34, 39]. For Danish Jersey, on the other hand, many of the currently observed QTL have been linked to lactose in milk. Four QTL (19_a, 19_b, 22_b, and 28_b) have been associated to lactose percentage in milk [18, 38]. In addition, the QTL on BTA 1 (1_b) harbouring *SLC37A1* and *AGPAT3*, and the QTL on BTA 5 (5_a) were both associated to alpha-lactalbumin in milk [27, 30]. Alpha-lactalbumin is a milk protein that plays a critical role in converting glucose into lactose [40]. Finally, the QTL 22_b was associated to wavenumbers, which interact with C-O exclusively. The QTL 22_b is harbours the gene *lactotransferrin* (*LTF*). The protein lactotransferrin is a selective antibacterial milk protein that is involved in the mucosal protection of the mammary gland, and possibly protects against mastitis [41, 42].

### Wavenumbers interacting with CO-N and N-H

The chemical bonds CO-N, and N-H are present in protein molecules. These chemical bonds interact with the infrared region that ranges from 1550 to 1500 cm^− 1^, and infrared region around 1600 cm^− 1^, respectively [1, 2]. These infrared regions are used for prediction of protein percentage in milk [1, 36]. The two groups of wavenumbers interacting with CO-N and N-H have many overlapping QTL, and therefore will be discussed together. Firstly, for both breeds, a strong association was observed between CO-N and N-H interacting wavenumbers and the *CSN* cluster on BTA 6 (6_b; Table 2). The *CSN* cluster has been associated to many traits related to protein in milk, such as protein percentage [18, 27, 30, 33], and protein composition [27, 30]. Secondly, a QTL on BTA 20 (20_b) was observed for both breeds. This QTL harbours the gene *ANKH*, which is strongly associated to alpha-lactalbumin, and it is expressed in mammary tissue [27]. Finally, one more QTL was observed for both breeds, which was located on BTA 17 (17_a). This QTL has been associated to alpha-S2-casein in milk [30].

For Danish Jersey, additional QTL were identified that have been associated to milk protein previously. Firstly, three QTL on BTA 3 (3_a), BTA 10 (10_b), and BTA 20 (20_a) have been associated to protein percentage in milk [30, 33]. Secondly, the QTL on BTA 11 (11_b) is located within a genomic region, which harbours several genes that control beta-lactoglobulin in milk [30, 43, 44]. Thirdly, the QTL no BTA 24 has been associated to beta-lactoglobulin previously as well [30]. Finally, like both the QTL on BTA 20 (20_b), and the QTL on BTA 5 (5_a) have been linked to alpha-lactalbumin [30].

### Wavenumbers interacting with –OH

Like C-O, the chemical bond -OH is abundantly present in sugar molecules. The chemical bond –OH interacts with wavenumbers in the infrared region that ranges from 1500 to 1250 cm^− 1^ [1, 2]. For Danish Holstein, wavenumbers from this infrared region were associated to the QTL on BTA 20 (20_b). This QTL harbours *ANKH*, which has been strongly associated to alpha-lactalbumin in milk [27]. Alpha-lactalbumin, as discussed earlier, has been described as a key player in lactose synthesis [40]. In Danish Jersey, the QTL explaining most variation was located on BTA 6 (6_b), which harbours the *CSN* cluster. Another QTL was located on BTA 1 (1_b) harbouring *SLC37A1* and *AGPAT3*, and has been associated to alpha-lactalbumin [27]. Two other QTL, which were positioned on BTA 19 (19_b) harbouring *FASN* and *CCDC57* and on BTA 28 (28_b), have been linked to lactose percentage in milk [18]. These two QTL have also been associated to wavenumbers surrounding wavenumber 1299 cm^− 1^ [18].

#### Breed differences

Breed has an effect on milk composition [24, 45], FT-IR milk spectra [5, 23], and the heritability of FT-IR milk spectra [17, 20, 21, 46]. In the current study more QTL were observed for Danish Jersey than for Danish Holstein (Table 1). A reason for this observation could be that *DGAT1* explained more additive genetic variation in Danish Holstein than in Danish Jersey. The less dominant role of *DGAT1* for Danish Jersey could have allowed for smaller effects to be visible. This could have resulted in the seemingly more polygenic character of milk spectra in Danish Jersey. Differences in allele frequency for the *DGAT1* gene have been described before [20, 25]. The fact that not the same QTL were observed for both breeds could have been caused by differences in allele frequencies for SNPs between the two breeds, or even the complete absence of SNPs in one breed [25, 47]. When applying milk spectra directly for estimating breeding values of milk components, these breed differences in allele frequencies may cause reduced prediction accuracy, when predicting across breeds.

## Conclusion

The current study observed multiple QTL for FT-IR milk spectra. Different QTL were observed for wavenumbers interacting with different chemical bonds. Wavenumbers that interact with the same chemical bond were often associated to the same QTL, yet some QTL were observed for small subsets of wavenumbers. Different QTL were observed for Danish Holstein and Danish Jersey.

## Methods

### Study population

The study population consisted of 3274 Danish Holstein cows from 354 herds, and 3408 Danish Jersey cows from 175 herds. For Danish Holstein, 3001 cows were in their first parity, and 273 in their second. For Danish Jersey, 3125 cows were in their first parity, and 283 in their second. For Danish Holstein, 19,656 morning-milk records were provided. For Danish Jersey, 20,228 morning milk records were provided. Cows had between one and twenty milk records with on average 32 days between records. Milk records were collected from October 1st 2015 to September 30th 2016. The year was split into summer, from April 1st 2016 through September 30th 2016, and winter, from October 1st 2015 through March 31st 2016. Milk records were collected from 1 through 400 days in milk (**DIM**). Obvious outlying milk records with a fat% > 8.0, or a protein% < 2.5 or > 5.0 in Danish Holstein, and a protein% > 5.5 in Danish Jersey were removed from the dataset.

Morning milk records were collected and provided by RYK (Aarhus N, Denmark), the Danish milk recording organization. Infrared spectral analysis was performed by Eurofins-Steins laboratory (Vejen, Denmark) with the MilkoScan FT+ (Foss, Hillerød, Denmark). Transmittance values for 1060 wavenumbers in the infrared region of 5008–925 cm^− 1^ were provided.

### Genotypes

The study population was genotyped with the EuroG10K custom SNP chip. The EuroG10k SNP chip is composed of two parts: (1) SNP from the BovineLD Genotyping BeadChip v.2 [48], and (2) a custom part of selected SNP from sequence data as part of 1000 Bull Genomes Project Run 4 [49] based on their functional annotation or based on GWAS results [50]. Genotypes were imputed from the EuroG10K custom SNP chip to the 50 K using BEAGLE 4 [51]. Reference populations for imputation consisted of 4000 cows for Danish Holstein, and 4576 cows for Danish Jersey. Reference cows were genotyped on the Illumina 50 K BovineSNP50 v.2 BeadChip (Illumina Inc., San Diego, CA). Only autosomal SNPs which were present in both the Danish Holstein reference population and the Danish Jersey reference population were selected. During quality control, SNPs with more than 40% missing genotypes or with a minor allele frequency (MAF) of < 0.01 were excluded. After quality control, genotypes of Danish Holstein cows were imputed from 10,353 to 43,807 SNPs, and genotypes of Danish Jersey cows from 9749 to 39,235 SNPs. Median distance between SNPs was 41 kb for Danish Holstein, and 43 kb for Danish Jersey. All SNPs used for analysis are present on the Illumina BovineSNP50 v.2 BeadChip (Illumina Inc., San Diego, CA).

### Phenotypes

#### FT-IR Milk spectra

Transmittance values were provided for 1060 wavenumbers in the mid-infrared region of 5008–925 cm^− 1^. Wavenumbers in the infrared regions from 5008 to 3008 cm^− 1^, and from 1669 to 1623 cm^− 1^ interact with water molecules, and were excluded from the analysis. A total of 530 wavenumbers were left for further analysis.

#### Selection of wavenumbers

Selection of wavenumbers was done for each breed separately. Correlations between 530 wavenumbers corrected for season, parity, days in milk, and herd were calculated in R software [52]. The correlation matrix was used to make a heatmap, where axes were sorted in order of wavenumber from 3008 cm^− 1^ through 925 cm^− 1^ (Fig. 1). Blocks of strongly positively correlated neighbouring wavenumbers were defined by visual inspection of the heatmap. Within each block, correlation sums were calculated for each wavenumber individually, and the wavenumber with the highest correlation sum was selected for further analysis.

### Genetic analysis

#### Model description

Analysis of selected wavenumbers was done with the Bayz software package (http://www.bayz.biz/) [53]. We used the model:

1 $$ {y}_{ijkl}=\mu +{Parity}_i+{Season}_j+{\beta}_1{DIM}_{ijkl}+{\beta}_2{e}^{-0.05{DIM}_{ijkl}}+{Herd}_k+{CowA}_l+{CowPE}_l+{E}_{ijkl.} $$

Where *y*_*ijkl*_ is the transmittance value for one selected wavenumber; μ is mean transmittance value; *Parity*_i_ corrects for the fixed effect of parity (*i* = 1 or 2); *Season*_j_ corrects for the fixed effect of season during which the milk sample was collected (*j* = summer or winter); *β*_1_*DIM*_*ijkl*_ and ${\beta}_2{e}^{-0.05{DIM}_{ijkl}}$ correct for lactation stage (Wilmink function) [54], where *DIM*_*ijkl*_ is *dim*_*ijkl*_ /365 (*dim*_*ijkl*_ = 1…365). For all fixed effects and regressors, a uniform prior distribution was assumed, where ~ UNI(0,+∞); *Herd*_*k*_ is a random herd effect, for which a normal prior distribution was assumed, where *Herd* ~ N(0, ${\sigma}_{Herd}^2$); *CowPE*_*l*_ is a permanent environmental effect of cow *l*, for which a normal prior distribution was assumed, where *CowPE* ~ N(0, ${\sigma}_{PE}^2$); and *E*_*ijkl*_ is the residual variance, for which a normal prior distribution was assumed, where *E* ~ N(0, ${\sigma}_E^2$). *CowA*_*l*_ is the additive genetic effect of cow *l*, and was modeled using a hierarchical model to depend on SNP effects:

2 $$ {CowA}_l=\sum m amglm $$

Where *a*_*m*_ is the additive effect of SNP *m*; *g*_*lm*_ is the allele dosage for SNP *m* of cow *l.* Allele dosages were centred. For the additive genetic value, a normal prior distribution was assumed, where *CowA* ~ N(0, ${\sigma}_A^2$), and all SNP variance parameters had a uniform prior distribution ~ UNI(0,+∞).

A Metropolis-Hastings sampler was used, with 70,000 iterations, including a burn-in of 30,000 iterations.

For all selected wavenumbers, heritability was calculated as:

3 $$ {h}^2=\frac{\sigma_A^2}{\sigma_{Herd}^2+{\sigma}_A^2+{\sigma}_{PE}^2+{\sigma}_E^2} $$

Where ${\sigma}_A^2$ is the additive genetic variance; ${\sigma}_{Herd}^2$ is the variance explained by herd; ${\sigma}_{PE}^2$ is the permanent environmental variance; ${\sigma}_E^2$ is the residual variance. Wavenumbers with a heritability < 0.05 were excluded from further analyses.

#### Grouping SNPs

Within each chromosome, SNPs were divided into groups of 100 consecutive SNPs [55]. The grouping procedure was repeated five times for each chromosome, starting with counting at SNP 1, 21, 41, 61, or 81 on the chromosome. Between the five repeated procedures, SNP groups overlapped, yet SNP groups were never identical. Groups with < 80 SNPs were excluded from analysis.

For each group of 100 SNPs, variance of the genomic estimated breeding value was calculated with the gbayz function of Bayz software (http://www.bayz.biz/) [53]. Proportion of total additive genetic variance explained per SNP group was calculated as:

4 $$ \%{\sigma}_{A, ij}^2=\frac{\sigma_{gEBV, ij}^2}{\sigma_{A,i}^2}\ast 100\% $$

Where $\%{\sigma}_{A, ij}^2$ is the percentage of total additive genetic variance of selected wavenumber *i* explained by SNP group *j*; ${\sigma}_{gEBV, ij}^2$ is the variance of the genomic estimated breeding value for selected wavenumber *i* of SNP group *j*; and ${\sigma}_{A,i}^2$ is the total additive genetic variance for selected wavenumber *i*.

Visual inspection was done on Manhattan plots of $\%{\sigma}_{A, ij}^2$, where $\%{\sigma}_{A, ij}^2$ of a group was represented by the middle SNP as orientation point (Additional files 1 and 2). For each selected wavenumber, QTL were collected.

## Supplementary information

**Additional file 1.** Manhattan plots of % of explained additive genetic variation for Danish Holstein. Scale of y-axis runs from 0 to 1%. The horizontal line indicates the cut-off at 0.35%, which was used to define and select QTL.

**Additional file 2.** Manhattan plots of % of explained additive genetic variation for Danish Jersey. Scale of y-axis runs from 0 to 1%. The horizontal line indicates the cut-off at 0.35%, which was used to define and select QTL.

## Data Availability

The raw datasets that were used in the current study are not available to the public, but can be requested for on reasonable grounds from the responsible co-author (AJB, bart.buitenhuis@mbg.au.dk). SNPs that were used in the current study were present on the 50 k Bovine SNP array from Illumina. SNP names, and the position of SNPs can be found on: http://support.illumina.com/downloads.html. Gene annotation was performed for all SNPs in Ensemble (92), using the UMD3.1 assembly, and the Variant Effect Predictor function [56].

## Abbreviations

**BTA**: *Bos Taurus* autosome
**DIM**: Days in milk
**FA**(**s**): Fatty acid(s)
**FT-IR**: Fourier transform infrared
**GWAS**: Genome wide association study
**QTL**: Quantitative trait locus/loci
