# Supplementary figures and images for: Genome-wide association study on Fourier transform infrared milk spectra for two Danish dairy cattle breeds
Source: BMC Genet. 2020 Jan 31;21:9. doi: 10.1186/s12863-020-0810-4 (PMC6993354; doi:10.1186/s12863-020-0810-4)

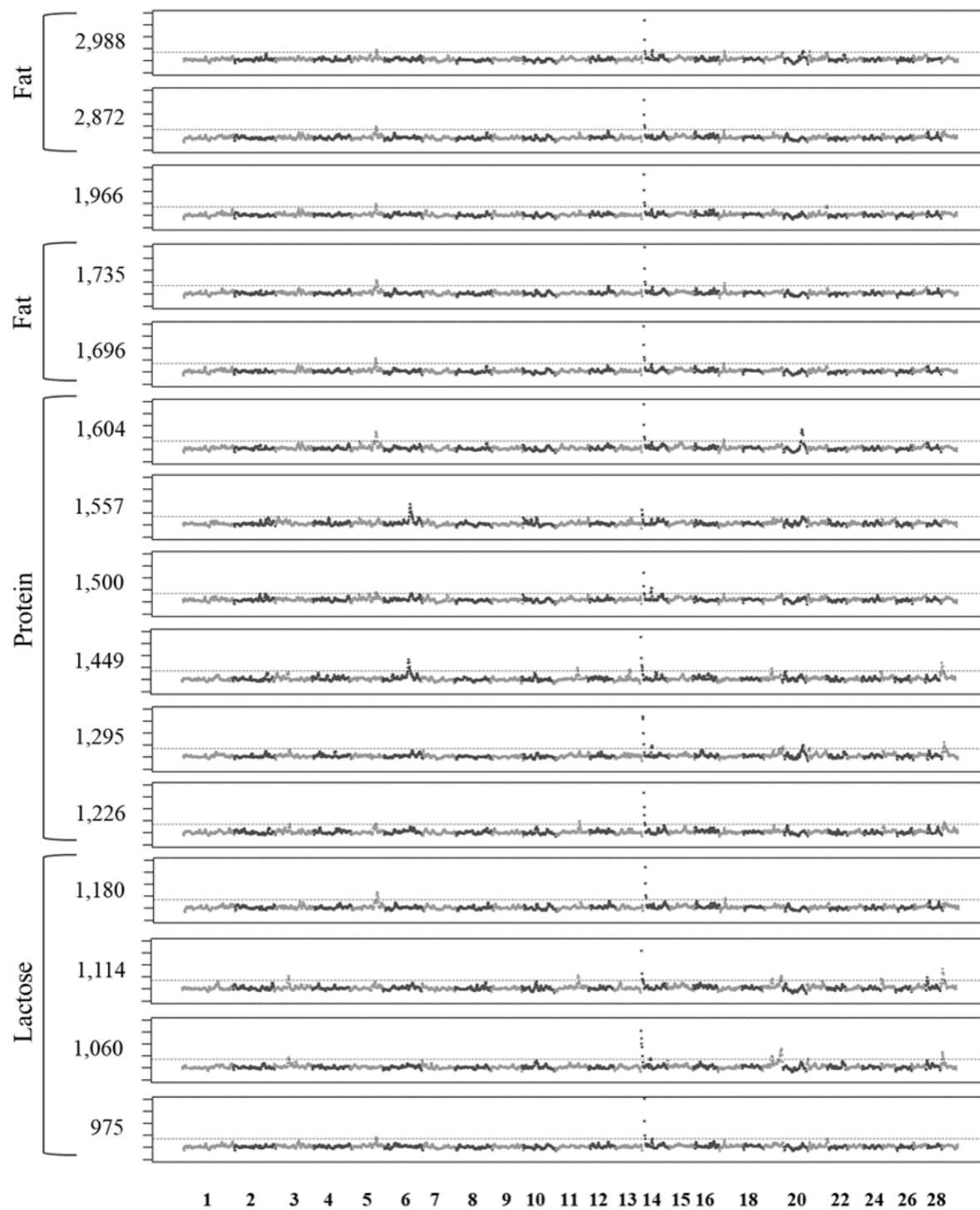

Supplement: Supplementary file 1 — Additional file 1. Manhattan plots of % of explained additive genetic variation for Danish Holstein. Scale of y-axis runs from 0 to 1%. The horizontal line indicates the cut-off at 0.35%, which was used to define and select QTL. [file 12863_2020_810_MOESM1_ESM.pdf]

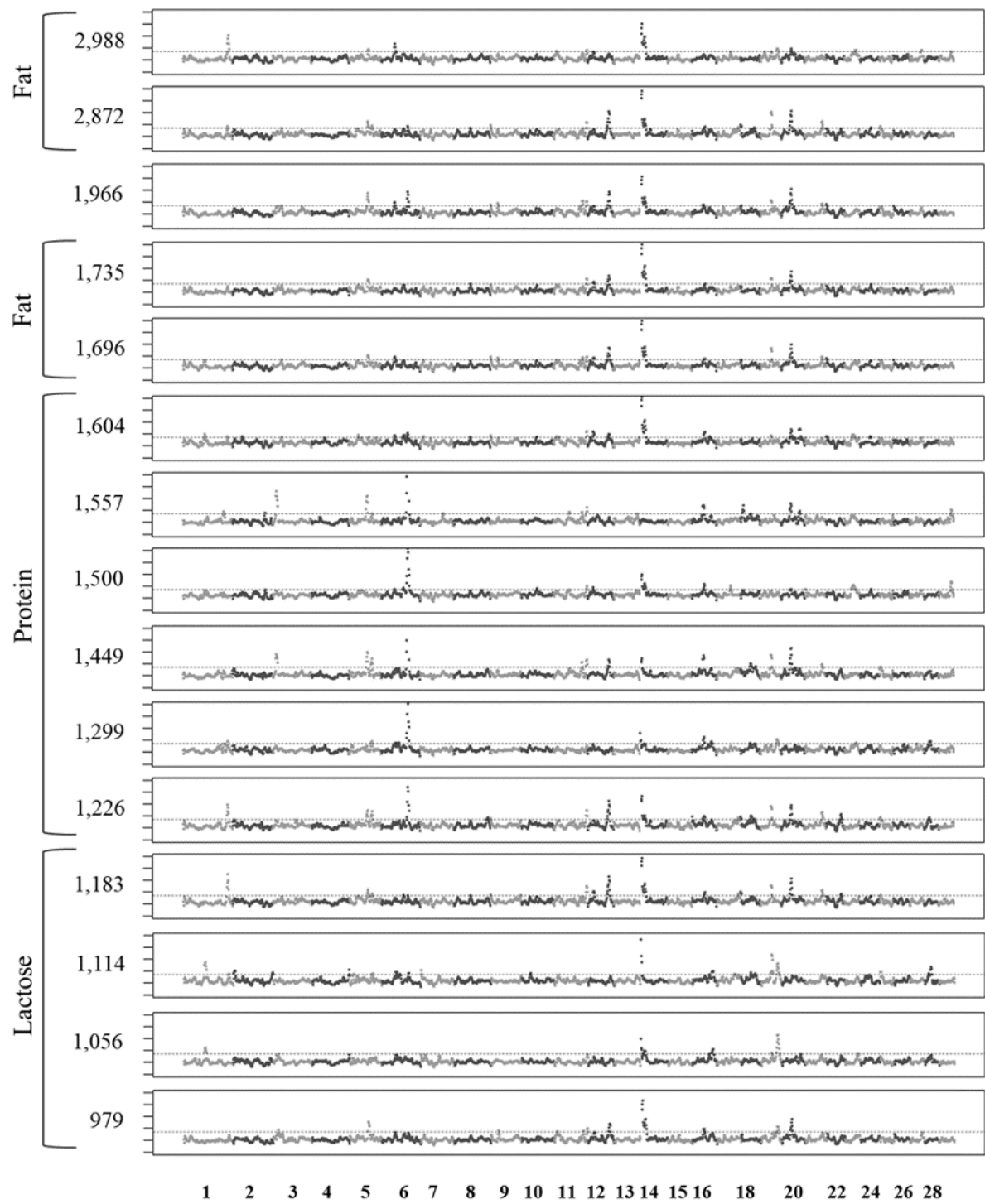

Supplement: Supplementary file 2 — Additional file 2. Manhattan plots of % of explained additive genetic variation for Danish Jersey. Scale of y-axis runs from 0 to 1%. The horizontal line indicates the cut-off at 0.35%, which was used to define and select QTL. [file 12863_2020_810_MOESM2_ESM.pdf]
